# Medicinal plants with promising antileishmanial activity in Ethiopia: A systematic review and meta-analysis

**DOI:** 10.1097/MD.0000000000038480

**Published:** 2024-05-31

**Authors:** Kassahun Misgana Worku, Birhanu Genanew Asfaw, Daniel Niguse Mamo, Yosef Haile, Habtie Tesfa, Mulugeta Aemero

**Affiliations:** a Department of Medical Laboratory Science, College of Medicine and Health Sciences, Arba Minch University, Arba Minch, Ethiopia; b Department of Health Informatics, School of Public Health, College of Medicine and Health Science, Arba Minch University, Arba Minch, Ethiopia; c Department of Public Health, School of Public Health, College of Medicine and Health Science, Arba Minch University, Arba Minch, Ethiopia; d Department of Medical Parasitology, School of Biomedical and Laboratory Sciences, College of Medicine and Health Sciences, University of Gondar, Gondar, Ethiopia.

**Keywords:** Ethiopia, herbal extracts, *Leishmania*, medicinal plants, meta-analysis, natural products, systematic review

## Abstract

**Introduction:**

Toxicity and resistance to chemotherapy used to treat leishmaniasis are increasing. Research on natural plant compounds has revealed their antileishmanial effects on certain *Leishmania* organisms. This review aimed to estimate the pooled IC_50_ values of medicinal plants with promising antileishmanial activity in Ethiopia.

**Methods:**

A systematic literature search was conducted using Science Direct, PubMed, Cochrane Library, and Google Scholar to locate potential studies. Studies published in peer-reviewed journals and gray literature in university repositories before April 1, 2022, which included a full-length study reporting the half-maximal inhibitory concentration (IC_50_) of Ethiopian medicinal plants that were written in English were included. Conference proceedings, review articles, letters to the editor, and correspondence were excluded. The quality of the included studies was assessed using the GIVIMP critical appraisal tools. Heterogeneity between studies was verified using Cochrane *Q* test statistics and *I*^2^ test statistics, and the effects were checked using Egger statistical test at a level of significance. A random-effects model was used to estimate the pooled IC_50_ of the medicinal plants.

**Results:**

Six articles that were conducted in Ethiopia that fulfilled the inclusion criteria, with a total of 62 in vitro experiments, were reviewed. The aggregated mean IC_50_ for medicinal plants in Ethiopia was 16.80 (95% CI: 12.44, 21.16) and 13.81 (95% CI: 13.12, 14.50) µg/mL for antipromastigote and antiamastigote activity, respectively. Aqueous was the significant preparation with IC_50_ of 0.53 (0.34, 0.73) µg/mL against promastigote and 0.98 (0.20, 1.76) µg/mL against the amastigote stage.

**Discussion:**

This review indicated that the pooled mean of IC_50_ for Ethiopian medicinal plants against promastigotes and amastigotes was relatively low and showed better efficacy. This strongly suggests the need to focus on antipromastigote and antiamastigote medicinal plants in Ethiopia for the development of antileishmanial drugs. It is necessary to identify their active components, and their potential toxic effects can lead to the production of well-tolerated and safe drugs for leishmaniasis. The high heterogeneity is the limitation of this study.

**Registration:**

The review has been registered at Prospero with identification number CRD42022343543.

## 1. Introduction

Leishmaniasis is an obligate intracellular protozoan disease transmitted through the bite of female blood-sucking sand flies belonging to the genus Phlebotomine.^[1]^ It is a neglected tropical disease (NTD) found in different parts of the world, including southern Europe, the tropics, and the subtropics.^[2]^ Based on the nature of clinical symptoms, leishmaniasis has 3 main forms: cutaneous leishmaniasis (CL), visceral leishmaniasis (VL) (also known as kala-azar), and mucocutaneous leishmaniasis (MCL), which are highly prevalent in developing countries and are related to malnutrition, poor housing, low income, weak immunity, and population displacement.^[1]^

According to a WHO report in 2020, of the 200 countries and territories that were reported to the WHO, 98 were considered endemic, and 6 of them had previously reported cases of leishmaniasis. Of those 200, 89 were thought to be CL endemic, 3 CL cases had previously been documented, 79 were thought to be VL endemic, and 5 VL cases had previously been published. 71 of the 200 individuals were classified by the WHO as having both CL and VL endemic conditions.^[3]^ Each year, 700,000 to be 700,000 to 1 million new cases are believed (50,000 to 90,000 for VL and 0.6 to 1 million for CL) and 20,000 to 30,000 fatalities.^[4,5]^

CL is known to cause a skin lesions that can last for months, and it is caused by *Leishmania major, Leishmania tropica, Leishmania aethiopica, Leishmania Mexicana, Leishmania braziliensis, Leishmania guyanensis, Leishmania panamensis, Leishmania peruviana*, and *Leishmania amazonensis*.^[5,6]^ VL, also known as kala-azar, is caused by species of the *Leishmania donovani* complex, which mainly targets tissue macrophages in systemic organs, such as the spleen, liver, and bone marrow, and should be regarded as a state of long-term parasitism, since the parasites are not completely eradicated, but rather remain in skin macrophages for a lifetime.^[7,8]^

Leishmaniasis is broadly classified according to its location in the western and eastern hemispheres. In the western hemisphere, the disease is known as new-world leishmaniasis, and is found in some areas of Mexico, Central America, and South America. In the eastern hemisphere, the disease is known as old-world leishmaniasis and is found in certain parts of Asia, the Middle East, southern Europe (particularly the Mediterranean region), North Africa, and tropical regions of Africa. New-world and old-world leishmaniasis are caused by different species of *Leishmania.*^[9]^

Only 7 nations account for almost all VL cases in the world: 4 in Eastern Africa (Sudan, South Sudan, Ethiopia, and Kenya), 2 in Southeast Asia (India, Bangladesh), and Brazil, which accounts for nearly all cases in South America. An annual incidence of 202,000 to 389,100 cases is estimated, with a 20,000 to 40,000 annual case fatality rate and 3.3 million disability-adjusted life-years (DALYs). Owing to gaps in surveillance and underreporting, VL data have various limitations. The African region had a reporting rate of 38%, the Eastern Mediterranean region 78%, and both the Americas and European regions 100%.^[10–12]^

Leishmaniasis has also been reported in Ethiopia. Both CL and VL cause increasing health problems in the country.^[13]^ VL is highly established in the Metema and Humera Plains, Center of the Omo Plains, Aba Roba Center, and Weyito River valley in the South Nations Nationalities region. It has also been reported in the Moyale area, the Genale River basin, the Awash Valley of the Oromia regional state, the Afdera district of the Afar region, and the Liban zone of the Somali region.^[14]^ In endemic areas, more than 4500 cases are reported annually. The vectors were associated with red acacia and balanite trees in the north and termite hills in the south.^[15,16]^

In contrast, cutaneous leishmaniasis has been well known since 1913 and is endemic in most regions of the country, yet it is one of the countries’ neglected diseases.^[17]^ An estimated 50,000 cases of the 3 clinical variants of LCL, MCL, and DCL are reported every year, all of which are primarily caused by the parasite *L aethiopica*, which is found primarily in the highlands.^[18,19]^ Rock hyraxes serve as reservoir hosts, while sand flies, *Phlebotomus longipes*, and *Phlebotomus pedifer* serve as vectors.^[20]^

Pentavalent antimonials, pentamidine, amphotericin B, lipid formulations of amphotericin B, paromomycin, and miltefosine are currently used as antileishmanial medicines that target various metabolic pathways.^[21]^ Pentavalent antimonials, which have long formed the backbone of antileishmanial treatments, are among the first-line medications. Antimonial medications have been associated with significant complications, such as cardiotoxicity, pancreatitis, hepatotoxicity, nephrotoxicity, and localized pain with intramuscular administration.^[22]^ *Leishmania* infection is also treated with second-line medications such as pentamidine and amphotericin B. Although amphotericin B can cause acute toxicity and requires hospitalization, it also has a disadvantage in terms of high expenses. However, pentamidine is no longer used because of its resistance and toxicity.^[23]^ Miltefosine has been recommended for use as an anticancer agent for VL and CL. The efficiency of this medication when taken orally and the speed of healing are the main advantages. However, it has substantial limitations in the treatment of leishmaniasis owing to its teratogenic effects and prolonged duration, which could promote drug resistance.^[24]^ An antibiotic called paromomycin, an aminoglycoside-aminocyclitol, has been prescribed to treat CL and VL via topical and parenteral administration, respectively.^[25]^

Various publications support the relevance of identifying active biological fractions and, as a result, active components in plants utilized in traditional medicines, and this is currently a wide-open topic of research.^[26,27]^ Plants are the source of approximately one-third to half of all pharmaceutical drugs,^[28]^ and natural cures are becoming increasingly popular worldwide.^[29]^ Due to its accessibility and biomedical benefits, there is a large amount of interest in medicinal plants in Ethiopia.^[30]^

Many studies have revealed that the limited supply of existing drugs and their high costs pose challenges in the fight against leishmaniasis.^[31]^ Another problem that makes the management of leishmaniasis more difficult is the growth of resistance to medications, which requires the search for viable alternatives.^[32]^ Therefore, exploring possible control mechanisms is central to addressing these problems, with the final aim of identifying a novel drug that can help combat them. Although studies have been conducted on the antileishmanial effects of medicinal plants, they have presented inconsistent and inconclusive findings. Furthermore, there has not been a previously conducted systematic review and meta-analysis has estimated the pooled mean IC_50_ for antileishmanial medicinal plants in Ethiopia. Therefore, this systematic review and meta-analysis were designed to estimate the pooled mean IC_50_ for antileishmanial medicinal plants in Ethiopia using available evidence.

## 2. Methods

### 2.1. Reporting system and registration

We conducted this systematic review and meta-analysis based on the reporting system of the preferred reporting items for systematic review and meta-analysis (PRISMA).^[33]^ It has been registered in Prospero with an identification number of CRD42022343543, available at https://www.crd.york.ac.uk/prospero/#recordDetails.

### 2.2. Data sources

Data were collected from electronic databases, such as Science Direct, PubMed, Cochrane Library, and Google Scholar, which were used as sources of information for previously published literature. Grey literature such as thesis, technical reports, working papers, evaluation reports, conference proceedings, patents, and preprints, were also considered for the review. The search for articles published is not time-limited and includes all papers published until April 1, 2022. The search words were used separately and in combination with Boolean operators such as “OR” or “AND.” In this study, only works published in English were used.

### 2.3. Searching strategy

The searching terms used in PubMed were (“Traditional Medicine”) OR (“Plant Preparation”) OR (“Herbal Preparation”) OR (“Healing Plants”) OR (“Plant extract”) OR (“Pharmaceutical Plants”) OR (“Herbal medicine”) OR (“Medicinal plants”) AND (Leishmania) OR (antileishmaniasis) OR (“Antileishmanial activity”) OR (“leishmanicidal activity”) OR (“*L major*”) OR (“*L aethiopica*”) OR (“*L tropica*”) OR (“*L donovani*”) AND (Ethiopia). Additional filters, such as the language (English), were used. Furthermore, other publications were identified from references cited in important articles and manually searched to identify further pertinent studies.

### 2.4. Eligibility criteria

#### 2.4.1. Inclusion criteria

This study comprised of in vitro studies carried out on medicinal plants used in Ethiopia and studies conducted in Ethiopia or any locality in Ethiopia. Full-length studies reporting the half-maximal inhibitory concentration (IC_50_) of Ethiopian medicinal plants written in English were included. All relevant studies found until our search period of April 1, 2022, were included. Furthermore, gray literature or papers archived in university repositories and related research written in English were included.

#### 2.4.2. Exclusion criteria

Studies that failed to describe the IC_50_ of Ethiopian medicinal plants were excluded. Other exclusion criteria included duplicate studies, unavailable full texts, abstract-only papers without information on extracted data, conference proceedings, review articles, letters to the editor, and correspondence.

### 2.5. Study selection and quality assessment

In this review, the retrieved articles were imported into Endnote X9 to collect and organize the search results and to remove duplicate articles. The titles and abstracts of the articles were screened by the reviewers (KMW, BGA, DNM, YH, HT, and MA). The reviewers appraised the methodological quality of the included studies using guidance document on good in vitro method practices (GIVIMP) critical appraisal tools.^[34,35]^ The tools consist of items to assess internal and external validity. Each item was used carefully to assess the methodological quality of the included studies. A value of 1 or 0 was given for each study according to the GIVIMP critical appraisal tools. A value of one (1) was given for items that were clearly stated in the method, whereas a value of zero (0) was given for items that were not stated clearly in the method part of the research. Finally, the overall methodological quality of the included studies was calculated as percentage. Articles with methodical qualities of <50%, 50–75%, and > 75% were considered poor, good, and high quality, respectively.^[34,35]^

### 2.6. Outcome of interest

The outcome of this systematic review and meta-analysis was the pooled mean IC_50_ for both antipromastigote and antiamastigote activities of Ethiopian medicinal plants.

### 2.7. Data extraction

The authors reviewed all the important parameters extracted from each study (KMW, BGA, DNM, YH, HT, and MA) using Microsoft Excel. For each study, the following items were extracted for analysis: authors, year of publication, parasite species, type of solvent used, extraction type, part of the plant used for extraction, exposure time, scientific names of the plants, family names of the plants, IC_50_, standard deviation, and standard error.

### 2.8. Statistical analysis

STATA 16 (STATA Corp, College Station, TX, USA) was used for the data analysis. The extracted data were entered into Excel and exported to STATA 16 for further analysis. The mean and 95% confidence intervals of the IC_50_ values were calculated for each study to estimate the pooled mean of the effects of herbal extracts on *Leishmania* spp. in Ethiopia. Random-effect model meta-analysis was used to estimate the pooled effect size and effect of each study with their confidence intervals. The degree of heterogeneity among the studies included in the meta-analysis was quantified using Higgins *I*^2^ statistics.^[36]^ If the upper limits of the *I*^2^ value were 25%, 50%, and 75%, it was assumed that they showed low, medium, or high heterogeneity, respectively.^[36]^ Subgroup analysis based on family name, preparation, parasite species, and parts used was used to resolve the occurrence of high heterogeneity in the included studies. Funnel plot analysis and Egger-weighted regression tests were performed to detect publication bias. A *P* value of < .05 in Egger test was considered evidence of statistically significant publication bias.^[37]^

### 2.9. Ethics approval

Ethical approval and consent to participate in this study are not applicable, because, as stated in the title, it is a systematic review and meta-analysis with no direct participation of study subjects as a primary study.

## 3. Results

### 3.1. Literature search and identified results

**Figure 1.**
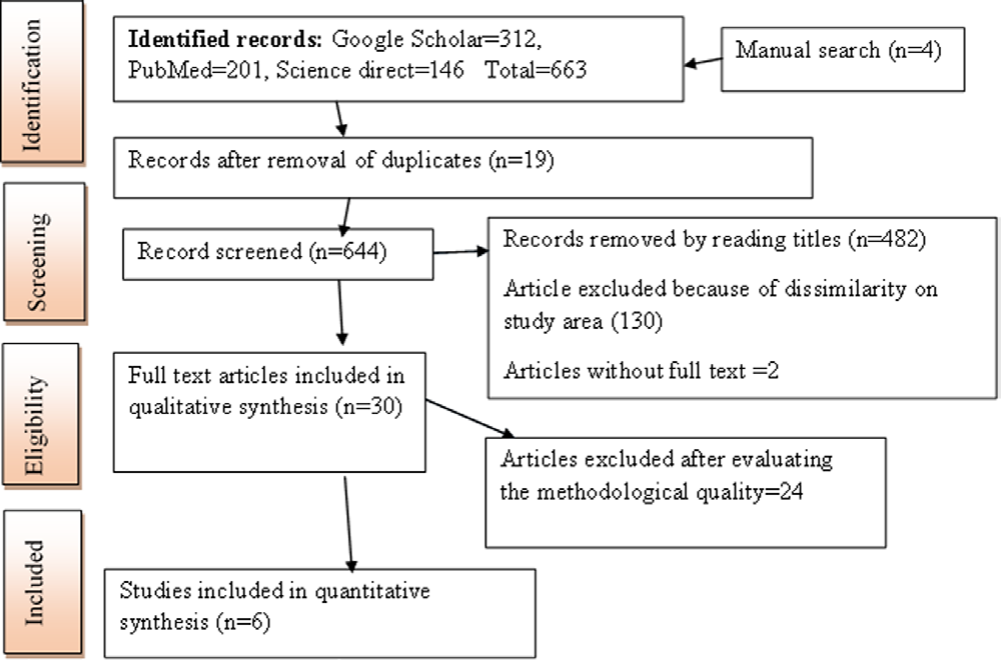
PRISMA flow diagram of article selection for systematic review and meta-analysis of medicinal plants with promising antileishmanial activity in Ethiopia. PRISMA = preferred reporting items for systematic review and meta-analysis.

A total of 663 studies were identified through a database literature search including manual search. Nineteen duplicate articles were excluded. In total, 644 articles were screened. Of these, 482 studies were removed after reading their titles, 130 studies were removed due to dissimilarity in the study area, and 17 studies were not included because of paper quality issues. After excluding nonrelevant articles, 6 full texts were identified and used for the final qualitative and quantitative analyses (Fig. 1).^[38–43]^

### 3.2. Description of included studies

In this review of 663 studies, 6 articles with 62 in vitro experiments met our eligibility criteria and were included in our review (Table 1). The extracted data comprised 10 types of plants tested against 3 species of *Leishmania*, their families, extraction methods, IC_50_ values, and *Leishmania* species. Thirty-six experiments were conducted on the antipromastigote activity of medicinal plants that contained 10 types of plants tested against 3 species of *Leishmania (L aethiopica, L donovani*, and *L major*). Twenty-six experiments were conducted on the antiamastigote activity of medicinal plants containing 8 plant species tested against 2 species of *Leishmania (L aethiopica* and *L donovani*). The methodological quality of the included studies was assessed using the GIVIMP critical appraisal tools.

**Table 1:** Summary of included studies in this systematic review and meta-analysis.

Author/Yr	Botanical name	Family name	Preparation	Products used	Part used	Species tested	Exp. time	IC_50_ (µg/mL) promastigote	IC_50_ (µg/mL) Amastigote
Sirak et al^[38]^	*Ranunculus multifidus*	Ranunculaceae	Methanol	Leaves	Leaves	*L aethiopica*	72 h	14.92 ± 0.55	17.49 ± 0.298
						*L donovani*		22.12 ± 0.56	19.325 ± 0.24
			Aqueous			*L aethiopica*		0.49 ± 0.004	1.49 ± 0.004
						*L donovani*		0.984 ± 0.028	1.814 ± 0.028
				Anemonin		*L aethiopica*		0.257 ± 0.007 (1.33)^a^	0.239 ± 0.014 (1.24)^a^
						*L donovani*		0.303 ± 0.304 (1.58)^a^	0.368 ± 0.024 (1.91)^a^
Abeje et al^[39]^	*Aloe calidophila Reynolds*	Asphodelaceae	Methanol	Leaf: Latex	Leaf: Latex	*L aethiopica*	72 h	64.05 ± 2.29	–
						*L major*		82.29 ± 3.62	–
			Methanol: Chloroform	Aloinoside		*L aethiopica*		1.76 ± 0.30	–
						*L major*		2.09 ± 0.21	–
				Aloin		*L aethiopica*		4.18 ± 0.45	–
						*L major*		7.16 ± 0.31	–
				Microdontin		*L aethiopica*		6.32 ± 0.79	–
						*L major*		8.85 ± 2.49	–
Chemeda et al^[40]^	*Aloe rugosifolia*	Asphodelaceae	Methanol	Leaf: Latex	Leaves	*L aethiopica*	72 h	24.50 ± 0.24	–
						*L donovani*		31.21 ± 0.01	–
			Methanol: Chloroform	Plicataloside		*L aethiopica*		14.22 ± 0.41 (27.66 ± 0.8)^b^	–
						*L donovani*		18.86 ± 0.03 (36.69 ± 0.06)^b^	–
Nigatu et al^[41]^	*Discopodium peninervium*	Solanaceae	Methanol	Leaves	Leaves	*L aethiopica*	72 h	13.38 ± 1.20	15.13 ± 2.31
						*L donovani*		63.62 ± 1.84	71.52 ± 0.32
	*Ferula communis*	Apiaceae	Methanol	Roots	Roots	*L aethiopica*		11.38 ± 0.55	14.32 ± 0.54
						*L donovani*		23.41 ± 2.32	31.12 ± 0.19
	*Otostegia integrifolia*	Lamiaceae	Methanol	Leaves	Leaves	*L aethiopica*		13.03 ± 1.29	16.84 ± 0.65
						*L donovani*		17.24 ± 0.87	14.55 ± 0.38
	*Urtica simensis*	Urticaceae	Methanol		Leaves	*L aethiopica*		63.78 ± 0.89	82.37 ± 1.63
						*L donovani*		44.37 ± 1.61	47.30 ± 1.08
Tewabe et al^[42]^	*Aloe macrocarpa*	Asphodelaceae	Methanol	Leaf: Latex	Leaves	*L aethiopica*	72h	1.90 ± 1.01	1.60 ± 0.01
						*L donovani*		1.92 ± 0.42	1.74 ± 0.21
			Methanol: Chloroform	Aloin A/B		*L aethiopica*		3.94 ± 0.33 (9.4)^b^	2.80 ± 1.02 (6.7)^b^
						*L donovani*		5.06 ± 0.22 (12.1)^b^	2.92 ± 0.15 (7.0)^b^
				Aloe-emodin		*L aethiopica*		2.81 ± 0.43 (10.4)^b^	2.23 ± 0.33 (8.3)^b^
						*L donovani*		2.85 ± 0.5 (10.6)^b^	2.75 ± 0.03 (10.2)^b^
				Rhein		*L aethiopica*		1.91 ± 0.61 (6.7)^b^	1.01 ± 0.22 (3.6)^b^
						*L donovani*		2.07 ± 0.11 (7.3)^b^	1.17 ± 0.42 (4.1)^b^
Habtemariam^[43]^	*Premna schimperi*	Lamiaceae	Ethanol	Diterpenes 4	Aerial parts	*L aethiopica*	72h	18. 33 ± 1.05	4.52 ± 0.97
	*Premna oligotricha*			Diterpenes 1				-	4.12 ± 0.28
				Diterpenes 2				11.67 ± 0.88	1.08 ± 0.25
				Diterpenes 3				-	19.2 ± 2.24

a Indicate concentration in nanomolar (nM).

b Indicates concentration in micromolar (µM).

### 3.3. Antileishmanial activity of Ethiopian medicinal plants

#### 3.3.1. Antipromastigote activity of Ethiopian medicinal plants

**Figure 2.**
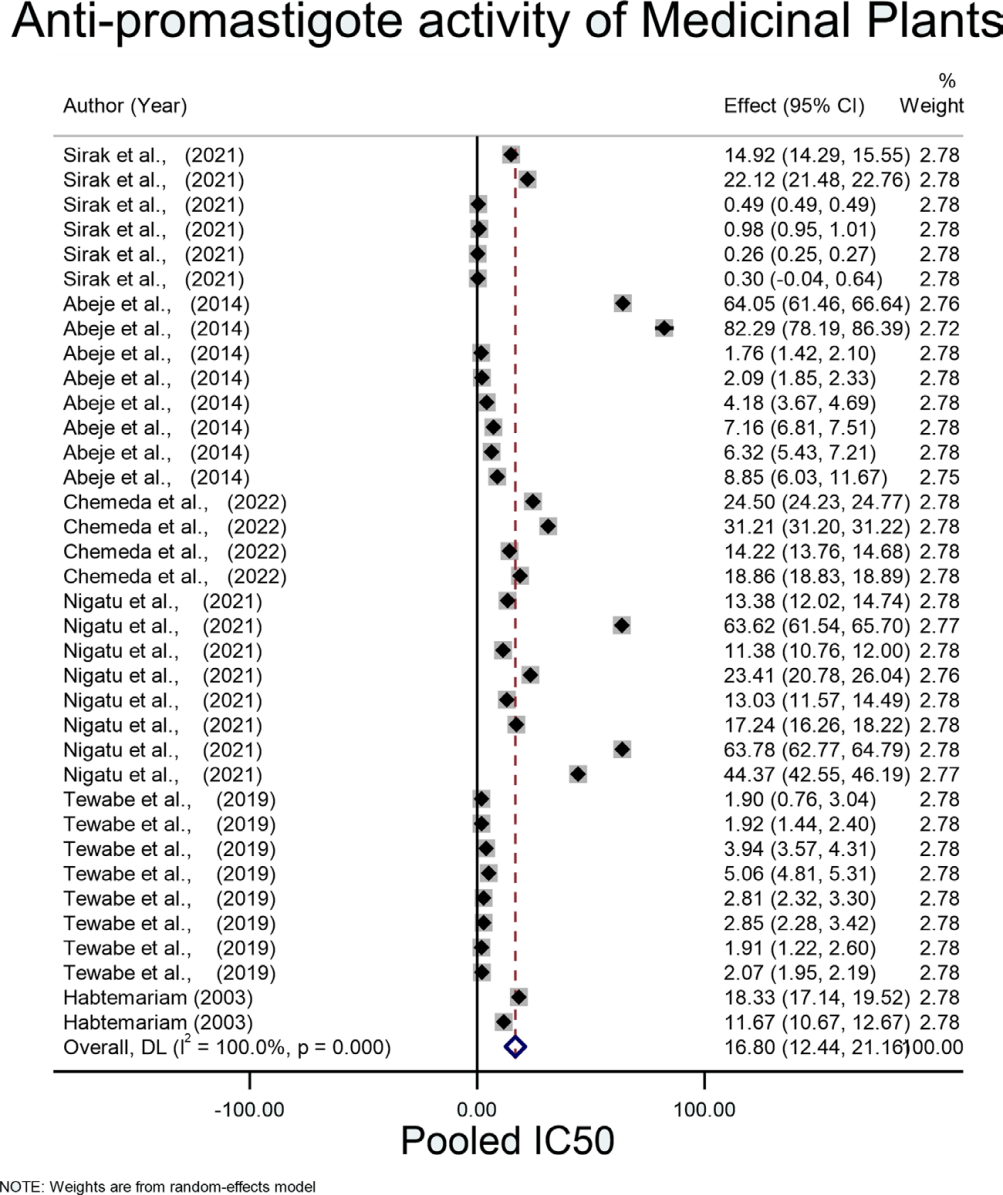
Pooled IC_50_ for antipromastigote activity of medicinal plants.

The pooled mean of IC_50_ using the fixed-effect model was 3.890 (95% CI: 3.887, 3.894) µg/mL with an *I* square value of 100% (*P* = .001). Because significant heterogeneity was observed in the fixed effects model, the Der Simonian-Laird random-effects model was used. The pooled mean of IC_50_ for antipromastigote medicinal plants in Ethiopia was 16.80 (95% CI: 12.44, 21.16) µg/mL (Fig. 2).

##### 3.3.1.1. *Subgroup analysis for pooled IC*_*50*_ *of antipromastigotes*

Subgroup analysis by family name, preparation, parts used, and species tested was done (Table 2).

**Table 2:** Subgroup analysis for the pooled IC_50_ from antipromastigote of Ethiopian medicinal plants.

Subgroup analysis by		Included studies	IC_50_ (95% CI) (µg/mL)	Heterogeneity (*I*^2^, *P* value)
Family name	Ranunculaceae	6	4.26 (3.97, 4.55)	100%, <.001
	Asphodelaceae	20	14.25 (9.43, 19.08)	100%, <.001
	Solanaceae	2	38.49 (−0.74, 87.73)	99.9%, <.001
	Apiaceae	2	17.33 (5.54, 29.11)	98.7%, <.001
	Lamiaceae	4	15.07 (11.81, 18.34)	97.0%, <.001
	Urticaceae	2	54.09 (35.07, 73.11)	99.7%, <.001
Preparation	Methanol	16	30.70 (24.66, 36.75)	100%, <.001
	Aqueous	4	0.53 (0.34, 0.73)	99.9%, <.001
	Methanol: Chloroform	14	5.86 (0.18, 11.54)	100%, <.001
	Ethanol	2	14.99 (8.47, 21.52)	98.6%, <.001
Species tested	*L aethiopica*	19	11.77 (11.36 12.17)	100%, <.001
	*L donovani*	13	17.98 (8.93, 27.03)	100%, <.001
	*L major*	4	23.98 (17.30, 30.65)	99.8%, < .001
Parts used	Leaves	24	15.22 (9.88, 20.56)	100%, <.001
	Leaf latex	8	21.18 (16.80, 25.57)	99.8%, <.001
	Roots	2	17.33 (5.54, 29.11)	98.7%, <.001
	Aerial parts	2	14.99 (8.47, 21.52)	98.6%, <.001

##### 3.3.1.2. Heterogeneity and publication bias

**Figure 3.**
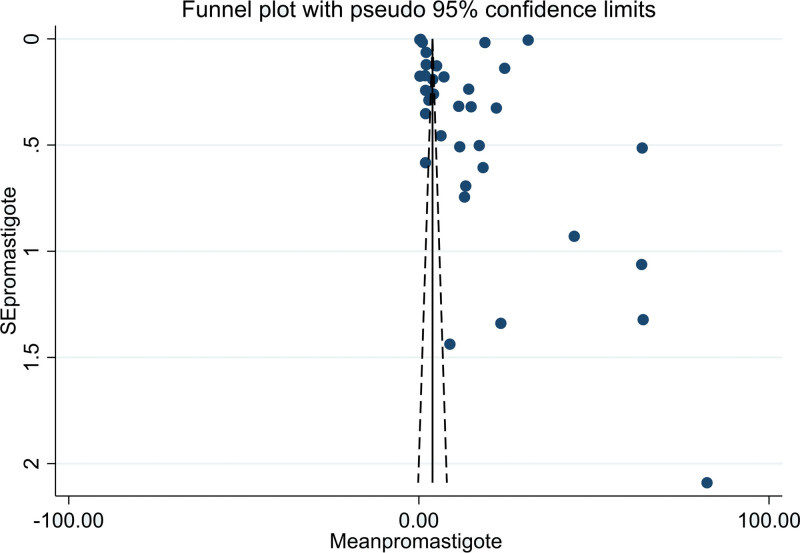
Funnel plot of included studies to determine the pooled antipromastigote of Ethiopian medicinal plants.

The heterogeneity of the included studies was high according to Higgins *I* square statistics (95%; *P* > .05). The included studies were visually assessed for potential publication bias by using funnel plots (Fig. 3) and Egger statistics (Table 3). In this review, the funnel plots of the included studies were asymmetrical. In addition, the Egger weighted regression statistics showed that (*P* > .05) (in this case, *P* = .458) indicated that there was no publication bias.

**Table 3:** Egger test of the included studies for the determination of the pooled IC_50_ of antipromastigote of Ethiopian medicinal plants.

Egger test	95% CI				
Std-Eff	Coef.	Std. err.	*P* value	Lower	Upper
Slope	3.451738	1.72801	.054	−0.0600005	6.963476
Bias	115.8285	154.1774	.458	−197.4977	429.1548

#### 3.3.2. Antiamastigote activity of Ethiopian medicinal plants

**Figure 4.**
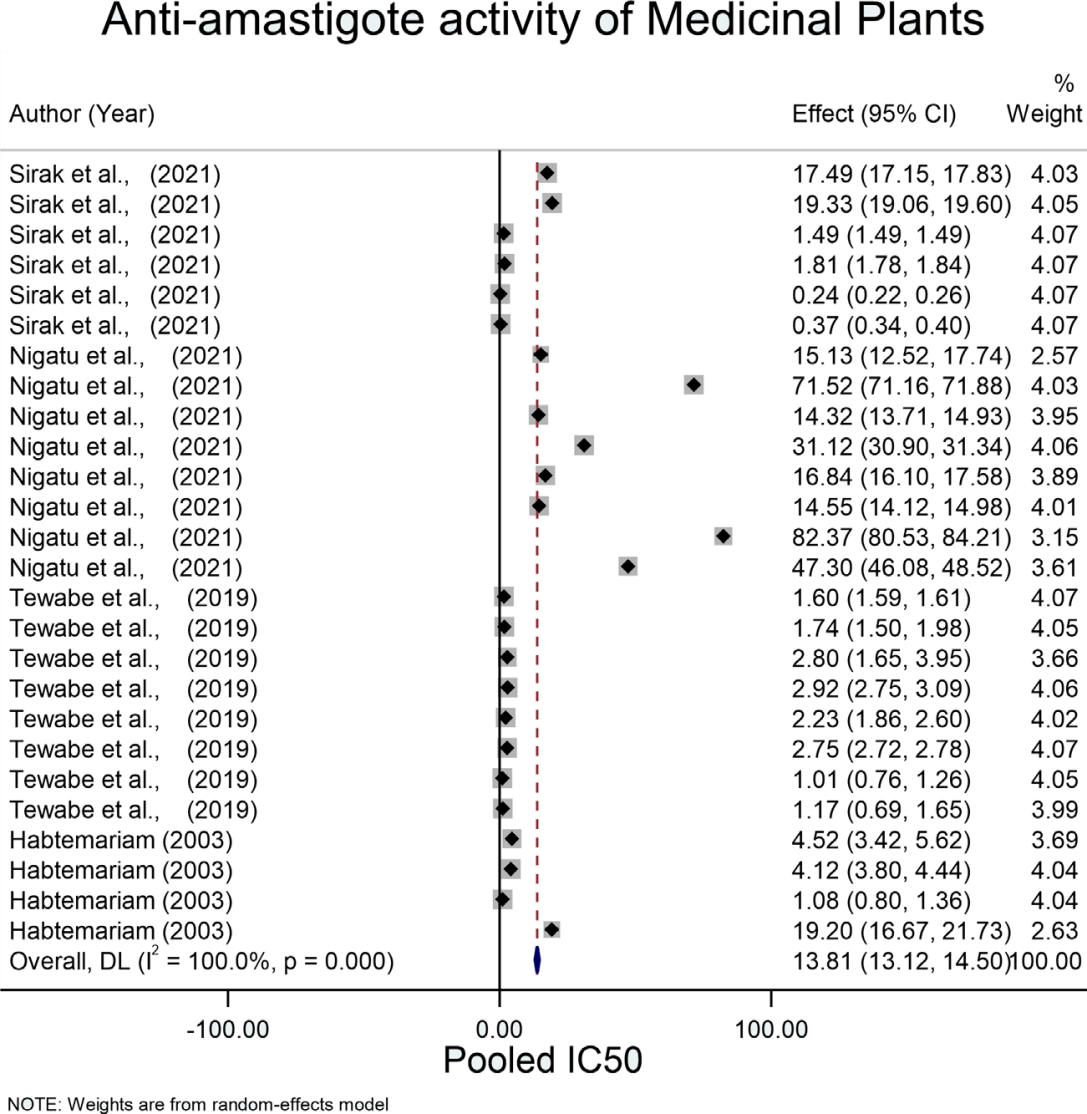
Pooled IC_50_ for antiamastigote activity of medicinal plants.

The pooled mean of IC_50_ using the fixed-effect model was 1.452 (95% CI: 1.448, 1.456) µg/mL with an *I* square value of 100% (*P* = .001) (Fig. 4). Since significant heterogeneity was observed in the fixed effects model, the Der Simonian-Laird random-effects model was used. The pooled mean of IC_50_ for antiamastigote medicinal plants in Ethiopia was 13.814 µg/mL (95% CI: 13.123, 14.504).

##### 3.3.2.1. *Subgroup analysis for pooled IC*_*50*_ *of antiamastigotes*

Subgroup analysis by family name, preparation, parts used, and species tested was done (Table 4).

**Table 4:** Subgroup analysis for the pooled IC_50_ from antiamastigote of Ethiopian medicinal plants.

Subgroup analysis by		Included studies	IC_50_ (95% CI) (µg/mL)	Heterogeneity (*I *^2^, *P* value)
Family name	Ranunculaceae	6	6.71 (5.83, 7.59)	100.0%, <.001
	Solanaceae	2	43.34 (−11.92, 98.60)	99.9%, <.001
	Apiaceae	2	22.72 (6.26, 39.19)	100.0%, <.001
	Lamiaceae	6	10.00 (4.57, 15.43)	99.9%, <.001
	Urticaceae	2	64.83 (30.46, 99.20)	99.9%, <.001
	Asphodelaceae	8	2.00 (1.43, 2.58)	99.8%, <.001
Preparation	Methanol	12	27.77 (16.15, 39.39)	100.0%, <.001
	Aqueous	4	0.98 (0.20, 1.76)	100.0%, <.001
	Methanol:chloroform	6	2.12 (1.52, 2.72)	97.9%, <.001
	Ethanol	4	6.70 (3.90, 9.51)	99.2%, <.001
Species tested	*L aethiopica*	15	8.53 (8.10, 8.96)	100.0%, <.001
	*L donovani*	11	17.65 (14.46, 20.85)	100.0%, <.001
Parts used	Leaves	20	14.07 (13.39, 14.75)	100.0%, <.001
	Roots	2	22.72 (6.26, 39.19)	100.0%, <.001
	Aerial parts	4	6.70 (3.90, 9.51)	99.2%, <.001

##### 3.3.2.2. Heterogeneity and publication bias

**Figure 5.**
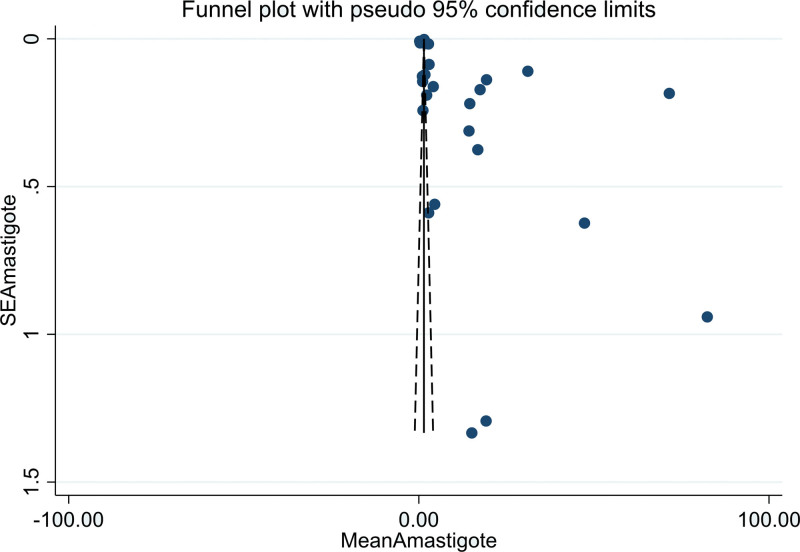
Funnel plot of included studies to determine the pooled antiamastigote of Ethiopian medicinal plants.

The heterogeneity of the included studies was high according to Higgins *I* square statistics (95%; *P* < .001). The included studies were visually assessed for potential publication bias by using funnel plots (Fig. 5) and Egger statistics (Table 5). The funnel plot of the included studies was asymmetric (Fig. 5). In addition, Egger weighted regression statistics showed that (*P* < .05) (in this case, *P* = .021). Thus, the test provides evidence for the presence of small study effects.

**Table 5:** Egger test of the included studies for the determination of the pooled IC_50_ of antiamastigote of Ethiopian medicinal plants.

Egger test	95% CI				
Std-Eff	Coef.	Std.err	*P* value	Lower	Upper
Slope	1.234242	0.2194386	.000	0.7813431	1.687141
Bias	52.71521	21.3369	.021	8.678009	96.75241

##### 3.3.2.3. Trim-and-fill Analysis

**Figure 6.**
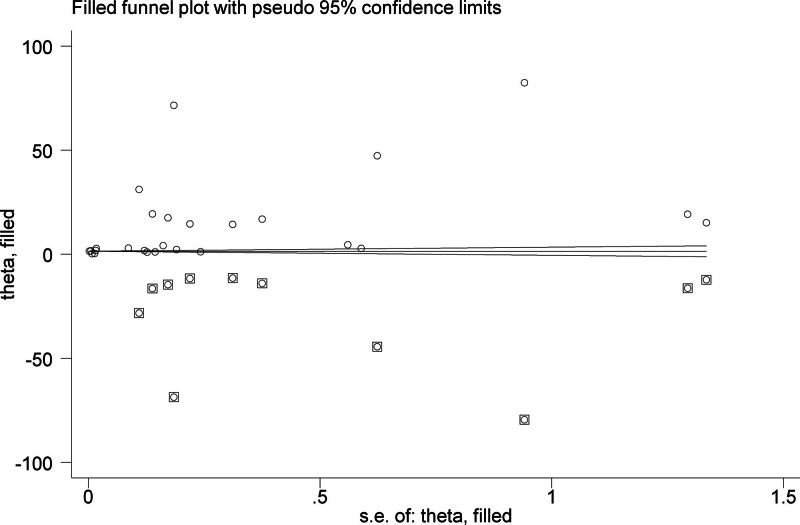
Trim-and-fill plot of pooled IC_50_ of antiamastigote medicinal plants.

As the *P* value was <.05 (0.021), there was a publication bias (small study effect). Therefore, trim-and-fill analysis was conducted (Fig. 6). After trim-and-fill analysis, the pooled estimated IC_50_ of antiamastigote medicinal plants was 1.66 (95% CI: 0.87–2.44) µg/mL (Fig. 6).

Number of studies trimmed to fit the model: 0.

Number of studies filled in to fit the model: 11.

## 4. Discussion

Leishmaniasis is a major health problem in impoverished nations, such as Ethiopia, due to malnutrition as a result of poverty, weak immunity, and the disease’s inability to respond to prescribed medications.^[44]^ Due to increased drug resistance, emerging cross-resistance, requirements for parenteral administration and/or length of treatment, lack of new drugs with novel mechanisms of action, and lack of effective vaccines, the effectiveness of current antileishmanial therapies is significantly declining^[8,45]^; as a result, innovative, economical, safe, effective, and easily administered medications are urgently needed.^[46]^ Alternative strategies, including the use of herbal plants, are being considered to reduce resistance in endemic areas. Antibacterial, antioxidant, anticancer, anti-inflammatory, and antileishmanial activities are among the pharmacological qualities of medicinal plant extracts.^[47]^

This study was conducted to determine the pooled IC_50_ of antipromastigotes and antiamastigotes from Ethiopian medicinal plants using 62 experiments. A wide range of plant extracts with antipromastigote and antiamastigote properties have been used in in vitro experiments. According to this review, among all medicinal plants, *Aloe* species (Asphodelaceae) are widely used, with recognized 46 species in Ethiopia.^[48]^ *Aloe* plants contain different types of phytochemicals and nutrients such as vitamins, minerals, enzymes, simple and complex polysaccharides, fatty acids, indoles, alkanes, pyrimidines, aldehydes, dicarboxylic acids, ketones, phenolic compounds, phytosterols, and alkaloids with potential biological and toxicological activities.^[49–51]^

In this study, the pooled IC_50_ of Ethiopian medicinal plants against promastigotes and amastigotes were 16.80 (95% CI: 12.44, 21.16) µg/mL and 13.81 (95% CI: 13.12, 14.50) µg/mL respectively which shows higher activity of Ethiopian medicinal plants against leishmaniasis (Figs. 2 and 4). When compared to the study from Iran, which has an IC_50_ of 456.64 (95% CI: 396.15, 517.12) µg/mL, it shows a high antileishmanial effect.^[52]^ This might be due to the variation in the plants containing secondary metabolites used against different *Leishmania* species.

The subgroup analysis by family name in this study indicated that Asphodelaceae (2.00 (1.43, 2.58)) µg/mL and Ranunculaceae (4.26 (3.97, 4.55) µg/mL) had lower IC_50_ values for antipromastigote and antiamastigote activities, respectively (Table 2 and Table 4). Conversely, the family Urticaceae has a higher IC_50_ of 64.83 (30.46, 99.20) µg/mL for antiamastigote activity and 54.09 (35.07, 73.11) µg/mL for antipromastigote activity. This is in agreement with studies conducted in India,^[53]^ Iran,^[54]^ Pakistan,^[55]^ and Kenya.^[56]^ This might be due to the similarity in the plant types used and the techniques applied for the extraction of the experimental plants.

In this study, the subgroup analysis by preparation of the extract indicated that the aqueous preparation has the lowest IC_50_ values for both stages of the *Leishmania* parasite, 0.53 (0.34, 0.73) µg/mL against the promastigote and 0.98 (0.20, 1.76) µg/mL against the amastigote stage. Similarly, the methanol preparation showed a higher IC_50_ value than the other preparations in the included studies. This finding is consistent with that of a study from Brazil^[57]^ and contradicts that of a study from Iran.^[58]^ This might be due to the solvent selection process used as well as the preparation technique used for the extraction of medicinal plants.

According to this study, a subgroup analysis of *Leishmania* species tested indicated that *L aethiopica, L donovani*, and *L major* have appropriately similar IC_50_ values against medicinal plants with antipromastigote activity, while antiamastigote activity was seen on *L aethiopica* and *L donovani*. On the other hand, while the aerial parts of the plant show a low IC_50_ value of 6.70 (3.90, 9.51) µg/mL against antiamastigote activity, the leaves, latex, roots, and aerial parts show almost similar IC_50_ values for antipromastigote activity.

This systematic review and meta-analysis showed that there was no publication bias based on the funnel plot and statistically confirmed the Egger test for the antipromastigote activity of Ethiopian medicinal plants included in the study (Table 3 and Fig. 3). Medicinal plants with antiamastigote activity showed a significant publication bias when checked by Egger test, which was solved by trim-and-fill analysis to handle publication bias (Table 5 and Fig. 6).

## 5. Strengths and limitations

The strengths of this systematic review and meta-analysis include an inclusive search using various databases, use of different searching strategies, critical appraisal of the methodological quality of the included studies using GIVIMP critical appraisal tools, and application of the PRISMA 2020 guidelines. This review is the first to provide consensus data on the Pooled IC_50_ of antileishmanial medicinal plants in Ethiopia. Although we performed subgroup analyses to minimize the effects of heterogeneity, the degree of heterogeneity among the reviewed articles was high. This might be due to differences in parasite species, type of solvent used, extraction type, part of the plant used for extraction, exposure time, and other unspecified variations. In addition, a strong publication bias was observed in the review because of the small study effect. Therefore, researchers and policymakers should consider the effects of heterogeneity and the results of a complete meta-analysis during the interpretation of the results.

## 6. Conclusion and recommendation

Finally, the results of the current study demonstrated that a variety of plant extracts had impacts on both the promastigote and amastigote stages of Leishmania, as well as intriguing antileishmanial abilities that were demonstrated in vitro. Therefore, it may be possible to use these extracts instead of synthetic medications. The available data indicate that phytotherapy has opened a broad and optimistic outlook for the development of novel, secure, and efficient leishmanicidal agents. The most advantageous formulation urgently required to confirm the efficacy of medicinal plants in the treatment of leishmaniasis is the current systematic investigation of the antileishmanial activity of medicinal plants, along with their toxicity, mechanism of action, and chemical properties for improvement. Overall, the present review offers useful knowledge on herbal combination therapy studies, experimental and clinical trials, and studies of natural compounds with antileishmanial activity. Additional clinical studies are required to develop safe and effective medicinal plant therapies. To create well-tolerated and secure leishmaniasis medications, it is vital to identify their active ingredients and their probable adverse consequences.

## Author contributions

**Conceptualization:** Kassahun Misgana Worku, Birhanu Genanew Asfaw, Habtie Tesfa, Mulugeta Aemero, Daniel Niguse Mamo, Yosef Haile.

**Data curation:** Kassahun Misgana Worku, Birhanu Genanew Asfaw, Habtie Tesfa, Mulugeta Aemero, Daniel Niguse Mamo.

**Formal analysis:** Kassahun Misgana Worku, Birhanu Genanew Asfaw, Habtie Tesfa, Mulugeta Aemero, Daniel Niguse Mamo, Yosef Haile.

**Methodology:** Kassahun Misgana Worku, Birhanu Genanew Asfaw, Habtie Tesfa, Mulugeta Aemero, Daniel Niguse Mamo, Yosef Haile.

**Software:** Kassahun Misgana Worku, Birhanu Genanew Asfaw, Habtie Tesfa, Mulugeta Aemero, Daniel Niguse Mamo, Yosef Haile.

**Supervision:** Kassahun Misgana Worku, Birhanu Genanew Asfaw, Habtie Tesfa, Mulugeta Aemero, Yosef Haile.

**Validation:** Kassahun Misgana Worku, Birhanu Genanew Asfaw, Habtie Tesfa, Mulugeta Aemero, Daniel Niguse Mamo, Yosef Haile.

**Visualization:** Kassahun Misgana Worku, Birhanu Genanew Asfaw, Habtie Tesfa, Mulugeta Aemero, Yosef Haile.

**Writing – original draft:** Kassahun Misgana Worku, Birhanu Genanew Asfaw, Habtie Tesfa, Mulugeta Aemero, Daniel Niguse Mamo, Yosef Haile.

**Writing – review & editing:** Kassahun Misgana Worku, Birhanu Genanew Asfaw, Habtie Tesfa, Mulugeta Aemero, Daniel Niguse Mamo, Yosef Haile.
